# Aging and Peripheral Nerve Injuries: Impaired Repair, Inflammaging Impact, and Regeneration Resistance

**DOI:** 10.3390/biomedicines14030636

**Published:** 2026-03-12

**Authors:** Xi Gu, Mengsi Lin, Yiming Xia, Xiangyu Cheng, Hongke Pan, Min Cai, Maorong Jiang, Dengbing Yao

**Affiliations:** 1 School of Life Sciences, Key Laboratory of Neuroregeneration of Jiangsu and Ministry of Education, Co-Innovation Center of Neuroregeneration, Nantong University, Nantong 226019, China; 2 Medical School of Nantong University, Nantong 226001, China; 3 Prenatal Screening and Diagnosis Center, Nantong Maternal and Child Health Care Hospital, Nantong 226001, China

**Keywords:** peripheral nerve regeneration, aging, Schwann cells, cellular senescence, inflammaging, oxidative stress, mitochondrial dysfunction, macrophage polarization, neuroinflammation, nerve repair

## Abstract

**Background**: Population aging is significantly altering the clinical conditions of peripheral nerve injury (PNI); however, the age-specific mechanisms that affect nerve regeneration remain unclear. Although the peripheral nervous system has the potential for regeneration, functional recovery after peripheral nerve injury is unsatisfactory in elderly people. The current research mainly focuses on young organisms, leaving a crucial gap in our understanding of how aging fundamentally alters the regenerative microenvironment and affects final therapeutic outcome. This review aims to integrate the latest evidence on aging-related changes in peripheral nerve repair and clarify the underlying mechanism of failed nerve regeneration in elderly people. **Summary**: An increasing amount of data indicates that aging not only delays the regenerative process but also significantly affects the nervous system’s microenvironment. In an aging environment, chronic low-level inflammation (known as “inflammaging”) caused by mitochondrial dysfunction, Schwann cell senescence, and abnormal macrophages impedes axon regeneration. Moreover, aging cells secrete pro-inflammatory mediators such as interleukin-6 and tumor necrosis factor-α, strengthening the paracrine aging process and establishing a positive feedback inflammatory cycle. We therefore integrated a metabolic–immune-aging framework to explain age-related regenerative resistance and emphasize the transformation barriers limiting clinical applications. **Conclusions:** Understanding the systems-level interactions within the aging nerve microenvironment is essential for developing age-tailored therapeutic strategies. Targeting metabolic dysfunction, immune dysregulation, and cellular senescence may offer new avenues for improving functional recovery in elderly patients with PNI.

## 1. Introduction

The peripheral nervous system (PNS) is the crucial communication network connecting the central nervous system (CNS) with peripheral organs; it is mainly responsible for functions such as sensory perception, motor coordination, and autonomous regulation. As a part of the nervous system, the PNS is mainly composed of nerves and nerve ganglia that connect the CNS with other parts of the body. Peripheral nerve injury (PNI) refers to varying degrees of damage to these nerves or nerve ganglia. Common types of PNI include avulsion, compression, laceration, or ischemic injury, as well as burns or frostbite [1]. At present, there are millions of cases of PNI worldwide each year [2]. In the United States, it is estimated that the annual incidence of PNI affects approximately 6 per 100,000 individuals, while in China, approximately 1,000,000 new cases of nerve injury occur each year [3,4,5]. Although the PNS’s regenerative potential is higher than that of CNS, its slow regeneration speed often leads to unsatisfactory structural and functional recovery after peripheral nerve injury, resulting in partial or complete loss of sensory, motor, and autonomic nerve functions [6,7,8,9,10,11]. Current data indicate that peripheral neuropathy, as one of the most common neurological diseases, has an annual incidence rate of approximately 77 cases per 100,000 people worldwide. Its prevalence varies from 1% to 12% across all age groups, even reaching 30% in the elderly population [12,13]. Moreover, the incidence of PNI-related tumor diseases peaks in the 90–94 age group, and the disease burden is highest in those aged 70–74 [14].

As population aging accelerates across the globe, the clinical burden of PNI is expected to increase significantly. Aging is not merely a temporal process but a complex biological phenomenon characterized by cellular function decline, metabolic changes, immune remodeling, and systemic low-level inflammation. These age-related changes significantly affect the tissue repair capabilities of various organ systems. In the nervous system, aging has long been regarded as the main risk factor for neurodegenerative diseases. However, its specific impact on peripheral nerve regeneration has received relatively little attention.

During aging, both the environment and function of the PNS undergo changes. Aging induces alterations, including but not limited to reduced neuroregenerative capacity, slowed nerve conduction velocity, and diminished neurological function [15]. These changes are caused by multiple factors, including immune system responses, metabolic factors, and gender differences [16,17]. Firstly, as one ages, the regenerative capacity of peripheral nerves significantly decreases, due to neuronal and supporting cell function degenerations [18,19,20]. For example, Schwann cells (SCs) in aged animals exhibit a reduced capacity to support regeneration, thereby limiting post-injury nerve recovery [21,22,23]. Secondly, aging can slow the conduction velocity of peripheral nerves, which is potentially linked to metabolic factors [24]. Studies have revealed that even when blood glucose levels are normal, low-grade inflammation and impaired glucose metabolism accelerate neurological function decline [25,26]. In addition, sex differences play a significant role in neurodegeneration [27,28]. Males experience a more pronounced decline in neurological function during later life than females [29]. As individuals age, an axon’s regenerative capacity significantly declines. This indicates that older individuals typically require a longer period for neural repair, which is mainly due to the reduced axon regeneration rate and formation of growth cones, as confirmed in rat and mouse models [30].

Despite these findings, the fundamental reason why aging impairs the peripheral nerve repair mechanism remains unclear. Existing studies often only focus on individual factors—such as oxidative stress, mitochondrial dysfunction, immune dysregulation, or Schwann cell senescence—without integrating these factors into a unified system model [19]. This review aims to address this deficiency by proposing an integrated framework from a systematic perspective: how upstream aging drivers (including mitochondrial dysfunction and chronic low-grade inflammation) interfere with the repair process of Schwann cells after nerve injury, causing abnormal polarization of macrophages and hindering myelin clearance, and ultimately altering the structural and metabolic “plasticity” of the regeneration microenvironment. Considering these mechanisms as parallel phenomena is not sufficient to fundamentally solve the problem, as these phenomena are interdependent and sequentially progressive, jointly determining the regeneration efficiency of elderly individuals. Based on this integrated perspective, we further propose several verifiable hypotheses and potential intervention targets, including: (1) regulating the Schwann cell phenotype to restore the repair ability of SCs; (2) improving the aging-related immune time-axis disorder phenomenon and regulating the dynamic response of macrophages and inflammatory reactions; and (3) combining metabolic regulation and strategies for clearing senescent cells to alleviate the degeneration of the neural microenvironment driven by inflammatory aging. By clarifying the connections and transformation nodes of these mechanisms, this article aims to provide a more structured theoretical basis for future basic and clinical research on aging-related PNI.

## 2. Literature Review

This review adopts a narrative review approach, aiming to synthesize current insights and translational research on the regeneration mechanisms of aging-related peripheral nerve injuries.

We conducted a comprehensive literature search using the PubMed database. The main search terms and their combinations used were as follows: peripheral nerve regeneration; aging; Schwann cells; cellular senescence; inflammaging; oxidative stress; mitochondrial dysfunction; macrophage polarization; neuroinflammation; nerve repair.

Boolean operators (AND/OR) were applied to refine the search and identify relevant studies at the intersection of aging, cell aging, inflammation, and nerve regeneration.

No time limit was set, and articles published up to [month, year] were included. Only studies published in English were included.

## 3. Effects of Aging on the Peripheral Nervous System

Aging not only involves a general decline in body function, but also profoundly affects the structural integrity and functional homeostasis of the PNS. Extensive research has indicated that age-related changes in peripheral nerves occur across multiple levels, including neurons, SCs, myelin structures, and the nervous system microenvironment. These changes ultimately lead to slowed nerve conduction, reduced regenerative capacity, and impaired sensory and motor functions.

At the structural level, aging is closely associated with a reduction in the number of peripheral nerve fibers and myelin degeneration. One morphological study revealed a significant decrease in sciatic nerve myelin thickness in aged animals, generally accompanied by segmental demyelination and axonal atrophy [31]. Proteomics analysis indicated that while the levels of major myelin structural proteins, such as myelin basic protein (MBP) and periaxin, remain relatively stable during aging, multiple extracellular matrix proteins, including collagen, are highly expressed. This suggests that mechanical properties at the myelin–matrix interface are altered, potentially affecting axonal signaling and the regenerative microenvironment [32].

At the cellular level, SCs act as key supportive and regenerative cells in the PNS, undergoing significant phenotypic and functional alterations during aging. The dedifferentiation capacity and remyelination efficiency of aged SCs are reduced, a phenomenon closely associated with impaired HGF/c-Met/c-Jun signaling pathway activation, which markedly slows nerve regeneration following injury [33]. In addition, the metabolic state of aged SCs shifts from supportive glucose metabolism to oxidative stress-driven metabolism, exacerbating inflammation and lipid peroxidation in the local neural microenvironment [34,35].

At the immune level, aging leads to localized immune cell composition and functional remodeling within nerves. In aging peripheral nerves, the number of macrophages significantly increases, exhibiting persistent activation and excessive phagocytosis. These phenotypes contribute to chronic inflammation and neurodegeneration [36,37]. “Inflammaging” not only disrupts nervous system homeostasis but also suppresses post-injury repair by interfering with the temporal dynamics and functional polarization of inflammatory reactions [38,39].

In addition, in peripheral nerve regeneration, gender differences have not been given sufficient attention. Increasing evidence indicates that there are significant post-nerve injury immune response differences between men and women, and these gender-related changes in macrophage activation and cytokine production can lead to different inflammatory characteristics [40,41]. Research has found that estrogen can exert neuroprotective and neuroregenerative effects by regulating the phenotype of SCs and promoting axon growth. Meanwhile, the reduction in androgens in elderly men may impair nerve regeneration [41,42]. What is crucial is that the aging characteristics interact with the endocrine system (such as menopause and male menopause), which may exacerbate inflammatory aging and reduce repair efficiency [42]. Clinical and epidemiological data further suggest sex-related differences in the prevalence and progression of peripheral neuropathies, particularly in elderly populations [43,44]. Overall, these research findings indicate that gender is not merely a biological variable, but also a crucial factor influencing the inflammatory environment and regenerative capacity of the peripheral nervous system after aging.

**Figure 1:**
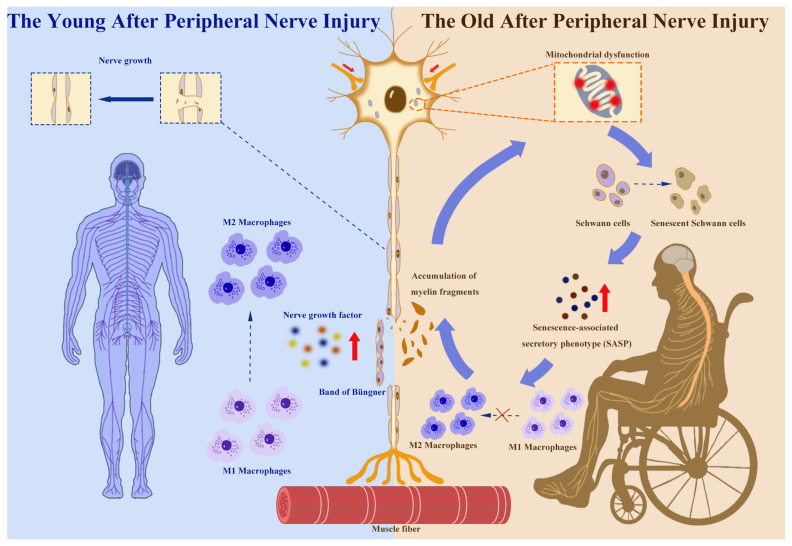
Effects of age on peripheral nerve regeneration after injury. Under normal circumstances, when a peripheral nerve is injured in an adult, Wallerian degeneration (WD) occurs at the injury site; macrophages are recruited there to phagocytose myelin debris and shift from the M1 to the M2 phenotype. Schwann cells (SCs) also proliferate to form Büngner bands and produce nerve growth factors that promote nerve repair. However, in older organisms, mitochondrial dysfunction and oxidative stress can promote the aging of SCs, generating senescence-associated secretory phenotype (SASP), interfering with macrophage polarization, making it difficult to clear the myelin debris at the damaged nerve sites, and the accumulated debris will further increase the oxidative stress response, ultimately leading to poor neural repair effects in the elderly body.

Furthermore, mitochondrial dysfunction is another core mechanism underlying age-related neurodegeneration (Figure 1). With aging, the accumulation of mitochondrial DNA mutations in nerve cells leads to reduced oxidative phosphorylation efficiency, resulting in insufficient energy supply and excessive reactive oxygen species (ROS) production. These metabolic disorders exacerbate axonal degeneration and myelin degradation, forming a vicious cycle. One animal study indicated that long-term caloric restriction significantly reduces age-related oxidative damage in the PNS (including protein oxidation and lipid peroxidation), thereby delaying the neural aging process at the molecular level [45]. This finding suggests that alleviating oxidative stress through metabolic regulation may offer a viable interventional strategy for age-related neurologic decline [45]. However, whether such metabolic interventions can also enhance nerve repair capacity in older adults following PNI remains unknown.

## 4. Repair Mechanisms Following Peripheral Nerve Injury

The PNS possesses a certain capacity for regeneration, enabling partial functional recovery following injury. However, this repair process is a complex biological event involving multiple stages and numerous cell types. It includes a series of dynamic processes such as axonal degeneration, inflammatory response, SC reprogramming, demyelination, and axonal regeneration [46].

### 4.1. Wallerian Degeneration

Wallerian degeneration is the initial step in nerve regeneration following injury, particularly after axonal disruption. First described by Waller in 1849, its core features include axonal fragmentation, myelin sheath fragmentation, and phagocytic clearance of debris (Figure 1) [47,48,49]. The speed of axonal and myelin degeneration depends on nerve fiber thickness, with thicker fibers undergoing Wallerian degeneration (WD) more rapidly. Although axonal changes are observed to slightly precede myelin changes, there is some overlap [50]. During WD, the myelin sheath, which is composed of SCs, undergoes myelinolysis and disintegration. However, very few SCs die during this process; instead, they begin to divide and proliferate 24 h later [46]. In addition, laminin, adhesion molecules, and other components on the surface of SCs and in their extracellular matrix (ECM) are highly expressed at the nerve injury site [51]. After myelin debris removal, proliferating SCs align in a band-like pattern parallel to the longitudinal axis of the nerve fiber within the basement membrane tube, forming cellular strands known as the Büngner band (Figure 1) [52]. During nerve regeneration, the Büngner band guides the extension of new axonal sprouts emerging from the proximal stump of the injured fiber toward their target structures [53]. On day 3 post-injury, large numbers of macrophages accumulate and, together with SCs, phagocytose and clear degenerated axons and myelin debris within the nerve basement membrane tube [54]. WD is thus an essential and indispensable change in the nerve regeneration process. It is not a passive degeneration but an active response mediated by programmed molecular pathways. The NAD^+^ metabolic pathway within axons plays a pivotal role. Following injury, the rapid degradation of nicotinamide mononucleotide adenylyltransferase 2 (NMNAT2) triggers Sterile α and TIR motif-containing protein 1 (SARM1) activation, leading to NAD^+^ depletion and energy collapse and ultimately resulting in axonal self-destruction [55]. In SARM1-knockout mouse models, axonal degeneration is significantly delayed, indicating that SARM1 serves as a crucial molecular switch for WD [56].

### 4.2. Myelin Debris Clearance and Immune Cell Response

Following WD, myelin debris within the injured nerve must be rapidly removed to avoid impeding regeneration. In the early stage, this process primarily relies on the autophagic and phagocytic functions of SCs [57]. SCs actively degrade the myelin sheath by upregulating autophagy-related genes, such as lysosomal-associated membrane protein 1 (LAMP1) and autophagy-related 7 (ATG7) [58]. Subsequently, circulating monocytes and macrophages infiltrate the injured site to complete debris clearance [59,60]. Macrophages phagocytose myelin and axonal fragments via antibody-dependent pinocytosis and Fc receptor-mediated pathways [61,62]. SCs actively promote macrophage recruitment by secreting inflammatory factors, such as interleukin (IL)-17B, while different cell types often depend on distinct receptor systems for debris clearance and inflammatory regulation [63]. TAM receptors (e.g., MER proto-oncogene, tyrosine kinase (MerTK) and Tyro3-Axl-Mer (Axl)) have been shown to play crucial roles in immunomodulation and phagocytosis within the nervous system, whereas macrophages predominantly depend on classical immune receptors to mediate their functions [64]. Macrophages play dual roles in this process: on one hand, they clear myelin debris through phagocytosis; on the other hand, they secrete growth factors and cytokines that directly or indirectly stimulate axonal regeneration [63,65]. Macrophage polarization state determines nerve regeneration quality: pro-inflammatory M1 macrophages clear debris in the early stage, while anti-inflammatory M2 macrophages promote axonal growth and myelin remodeling in the later stage (Figure 1) [66]. Furthermore, Toll-like receptors (TLRs) on SCs are involved in initiating SC activation and macrophage recruitment. TLR gene knockout (e.g., TLR2, TLR4) impedes myelin debris clearance, delays nerve regeneration, and changes the expression of myelin-related genes [67,68,69].

### 4.3. Regenerative Schwann Cell Reprogramming

Following nerve injury, SCs shift from myelinating to reparative states. This process is primarily driven by the transcription factor c-Jun, with its rapidly rising expression, inducing demyelination and the secretion of multiple neurotrophic factors (e.g., nerve growth factor (NGF), brain-derived neurotrophic factor (BDNF), and glial cell line-derived neurotrophic factor (GDNF)) (Figure 1) [70,71]. Reparative SCs also form Büngner bands, providing target structures for regenerating axons [48,52]. c-Jun knockout significantly impedes nerve regeneration, indicating that this transcriptional program serves as a core mechanism for nerve repair [72]. Moreover, reparative SCs release exosomes that are internalized by peripheral neurons to promote axonal regeneration [73].

### 4.4. Axonal Regeneration and Target Organ Reconstruction

Following debris clearance and SC reprogramming, axonal regeneration is initiated. Regenerating axons grow along the Büngner bands and rely on neurotrophic factors (e.g., NGF, Neurotrophin-3 (NT-3)) for directional guidance [74,75]. Nerve regeneration success is influenced by multiple factors, including intrinsic axonal growth capacity, ECM composition, inflammatory status, and vascular remodeling [76]. Newly formed blood vessels not only nourish axons but also provide a scaffold for SC migration. Notably, these mechanisms become significantly less efficient with age. In aged animals, incomplete nerve repair is attributed to reduced c-Jun expression in SCs, delayed macrophage-mediated debris clearance, and diminished angiogenesis [77,78].

## 5. How Aging Disrupts Regenerative Coordination

Aging-associated peripheral nerve regeneration disorder is not solely caused by cellular changes. This phenomenon constitutes a pathological network that is interconnected with mitochondrial dysfunction, Schwann cell senescence, and immune dysregulation.

As one ages, mitochondrial dysfunction becomes a crucial trigger factor in aging tissues. The accumulation of mitochondrial DNA damage, impaired oxidative phosphorylation function, and excessive reactive oxygen species (ROS) production all lead to metabolic stress responses, thereby promoting cellular aging in SCs and neurons [79]. At the same time, insufficient energy will impede axonal transport and cytoskeleton remodeling, thereby reducing the cell’s own regenerative capacity [80].

Mitochondrial stress further activates NF-κB-dependent inflammatory signaling and promotes the development of the senescence-associated secretory phenotype (SASP) [81]. The senescent SCs will secrete pro-inflammatory cytokines such as interleukin-6 and tumor necrosis factor-α. These factors will enhance cells’ aging response and maintain a chronic inflammatory state in the microenvironment of the damaged nerves [82,83]. This amplification of the inflammatory cycle model interferes with macrophages’ normal transformation process from a pro-inflammatory (M1-like) to a proregenerative (M2-like) phenotype [84].

If the polarization of macrophages is disrupted, this will further hinder cell debris, prolong inflammatory response duration, and increase oxidative stress. In turn, this will exacerbate mitochondrial damage and SC dysfunction [85]. Mitochondrial impairment, cellular senescence, and immune dysregulation form a self-reinforcing pathological circuit.

Simultaneously, aging inhibits the activation of gene programs related to neurons and SC regeneration [86]. c-Jun’s induction effect is weakened, the secretion of neurotrophic factors decreases, the components of the extracellular matrix change, and vascular remodeling is impaired. These factors collectively reduce the availability of the regenerative microenvironment [77]. These processes do not occur independently but rather intermingle with each other, jointly creating an unfavorable microenvironment that hinders nerve axon elongations, myelin sheath regeneration, and functional recovery.

Therefore, the inhibition of aging-induced neural regeneration should be understood as a systemic breakdown involving metabolism, inflammation, and changes in cell characteristics. Treatment strategies targeting a single pathway may not be very effective. Instead, in elderly people, multi-target or signal network-based intervention measures may need to be adopted to restore the regenerative capacity in the peripheral nervous system.

## 6. Clinical Implications of Aging in Peripheral Nerve Injury

Clinical studies consistently demonstrate that advanced age is associated with delayed and incomplete functional recovery after peripheral nerve injury [18]. Electrophysiological analysis indicates that compared to younger individuals, elderly patients exhibit reduced nerve conduction velocity and decreased compound muscle action potential after nerve repair surgery [18]. At the same time, the decline in age-related axonal regeneration capacity leads to an extended nerve regeneration time, increasing the possibility of irreversible motor endplate atrophy. Moreover, delayed nerve regeneration results in progressive muscle atrophy and fibrosis, further compromising functional prognosis [87]. Elderly patients’ sensory recovery is also impaired, possibly due to reduced density of regenerating sensory fibers and neurotrophic support [88]. In summary, these findings indicate that aging not only alters molecular mechanisms but also translates into clinically significant functional deficits.

In addition, in elderly patients, peripheral nerve regeneration is often affected by various age-related complications, such as diabetes, vascular diseases, and muscular atrophy [89]. The characteristics of diabetic neuropathy include microvascular dysfunction, reduced neural blood flow, and metabolic SC disorders, all of which can affect axonal regeneration [90]. Chronic hyperglycemia can also trigger oxidative stress and mitochondrial dysfunction, further exacerbating nerve injury response [91]. Similarly, systemic vascular diseases can lead to ischemia in the nerves and reduced nutrient supply, thereby limiting nerve regeneration [92]. Muscular atrophy is also a key factor; with age, muscle loss shortens the effective time for nerve reconnection and accelerates motor endplate degeneration [93]. In elderly patients, the nerve repair process must therefore be interpreted within a broader systemic context, rather than being regarded as an isolated event.

## 7. Potential Therapeutic Strategies for Regeneration in the Aging Nervous System

Given the complex alterations in peripheral nerve regeneration mechanisms during aging, multiple intervention strategies have been proposed in recent years. The primary therapeutic approaches and their mechanisms of action are summarized in Table 1.

### 7.1. Drug Therapy

Rapamycin is a macrolide drug produced by Streptomyces hygroscopicus, which was initially discovered as an antifungal agent. Recently, rapamycin has been found to inhibit the mechanistic target of mammalian target of rapamycin (mTOR) and extend the average lifespan of *yeast*, *Caenorhabditis elegans* (*C. elegans*), and *Drosophila melanogaster*; however, its effects in mammals require further exploration [94]. Rapamycin also possesses neuroprotective effects. Oral administration effectively improves cognitive function in aged mice and maintains blood–brain barrier integrity by preventing neuronal loss [95,96]. As described above, the PI3K-Akt-mTORC1 signaling pathway plays a crucial role in peripheral nerve regeneration following injury. As a typical mTOR inhibitor, rapamycin has a significant inhibitory effect on mTORC1. By inhibiting S6K1 (a ribosomal protein S6 kinase) and protein translation while increasing autophagy, mTORC1 can promote longevity [97]. However, long-term excessive administration of rapamycin inhibits mTORC2 expression, potentially leading to metabolic dysfunction and shortened lifespans in male mice, although the mechanism remains unclear [98].

Designed to selectively eliminate senescent cells, senolytics have recently been developed as a promising strategy for extending health and lifespan. Senescent cells typically increase several anti-apoptotic regulators, such as dependence receptors, PI3K/Akt, and BCL-2, which can together regulate cells’ anti-apoptotic ability [99]. Although senolytics such as dasatinib and quercetin have shown certain potential in preclinical models, their clinical translation for application in the elderly population still requires cautious consideration. Dasatinib may cause hematological suppression, hepatotoxicity, and fluid retention, which could limit its tolerance in frail elderly individuals [100,101]. Similarly, although calorie restriction can regulate metabolic and inflammatory pathways, it may exacerbate muscle atrophy, weakness, and malnutrition in the elderly, especially for those with other complications [102,103]. Additionally, polypharmacy and age-related pharmacokinetic changes increase the risk of drug interactions and adverse reactions in elderly patients [104]. Therefore, before safely applying these strategies to elderly patients with peripheral nerve damage, dose optimization, intermittent treatment regimens, and strict age-stratified clinical trials must be carried out.

### 7.2. Stem Cell Therapy

Multiple therapeutic approaches have been developed for aging or chronically denervated peripheral nerves, with mesenchymal stem cells (MSCs) being relatively widely used. MSCs have been identified as the most promising factor for promoting axonal regeneration and nerve repair after injury [105]. MSCs are self-renewing multipotent progenitor cells with the ability to promote neuroprotection and tissue repair. MSC-loaded artificial neural tissues can both promote nerve growth and modulate the microenvironment in the injured region. To date, MSCs have been used through various techniques in neural tissue engineering to enhance regeneration and repair following PNI. Primary or genetically modified MSCs have been used for regenerating liver, heart muscle, nerves, bone, tendons, and other connective tissues. Normal MSCs have a short survival time and low survival rate after implantation into the host injury site, whereas genetically modified MSCs can overcome these limitations. Through optimized selection of stem cells and delivery vectors, specific genes can be effectively introduced into MSCs. The protein expression capacity of genetically modified MSCs is validated in in vitro experiments, and selected genetically modified MSCs can be used to treat acquired and genetic diseases in clinical practice [106]. In the field of tissue engineering for nerve repair, genetic modification primarily aims to design target cells that can excessively release growth factors, chemotactic molecules, and adhesion molecules while suppressing defective gene expression. Genetically modified MSCs have been widely applied in neurological research. However, aging can influence both endogenous and exogenous stem cells. The proliferative potential of various stem cell niches in vivo declines with age. This reduction in the proliferative capacity of stem cells significantly impacts bodily maintenance. For example, cell cycle activity in the hematopoietic stem cells is reduced in old compared to young mice [107]. Similarly, numerous studies have observed age-related declines in the in vitro and in vivo functions of bone marrow-derived MSCs [108,109]. Furthermore, there are also some ethical and safety concerns regarding the clinical application of MSCs [110]. Addressing these concerns, the development of induced pluripotent stem cells circumvents ethical issues by generating pluripotent cells without destroying embryos. Consequently, research efforts have shifted toward utilizing induced pluripotent stem cells (iPSCs) as a source of SCs. This approach uses derived SCs to repair aged and damaged nerves. However, these stem cells have relatively low purity and yield [111]. Research on treating neurological disorders with stem cells has rapidly expanded since they were first discovered in 1980 and used to treat Parkinson’s disease (PD). Stem cells from diverse sources have been identified and utilized. Technological developments have also improved transplantation methods, making them safer and more reliable. However, many questions remain to be answered [112].

### 7.3. Gene Therapy

With the development of gene editing technologies, significant progress has been made in gene therapy targeting aging peripheral nerves. First, as the most prevalent technique, CRISPR-Cas9 is a powerful gene editing tool that precisely modifies specific gene sequences. In treating aging peripheral nerves, CRISPR-Cas9 is used to target genes associated with neural function. For example, it mediates genome editing and expression in induced pluripotent stem cells to repair aged nerves via derived neurons [113]. To explore the genetic and epigenetic basis of cellular aging, Wang et al. conducted a CRISPR-Cas9 screening and identified KAT7 (a histone acetyltransferase gene) as a driver of senescence in human mesenchymal progenitor cells [114]. Screening for and identifying suitable genes from functional genomes is thus crucial for developing antiaging interventions.

In addition, RNA interference technology (RNAi) regulates neural function by targeting specific mRNAs to inhibit the expression of specific genes. For example, RNAi can be used to knock down NF-κB, thereby inhibiting the expression of certain inflammation-related genes (such as tumor necrosis factor-α (TNF-α)), reducing neuroinflammation and cell death, and promoting nerve repair [115].

Epigenetic mechanisms, including DNA methylation, histone modifications, and non-coding RNAs, play essential roles throughout all stages of nervous system development [125]. Indeed, the nervous system is particularly susceptible to disruptions in epigenetic regulation. Modulating DNA methylation can influence gene expression, thereby affecting nerve cell development and function. These mechanisms also play significant roles in neurodegenerative diseases and neuroaging [116].

### 7.4. Physical Therapy and Rehabilitation

In addition to the above-mentioned approaches, physical therapy has also made significant strides in delaying and improving peripheral nerve aging. For example, low-intensity laser therapy can act on peripheral nerves through specific laser light wavelengths, promoting cellular energy metabolism and nerve regeneration. This method has been applied to treat nerve injuries and neuropathies, demonstrating potential protective effects on aging peripheral nerves [117].

Since the 1980s, numerous animal studies have shown that electrical stimulation therapy positively influences peripheral nerve recovery. In a rat femoral nerve model, continuous electrical stimulation at 20 Hz applied to the proximal nerve end shortened the axonal growth phase from 10 to 3 weeks [118,119]. Electrical stimulation approaches that change neuromuscular activity through current primarily include neuromuscular electrical stimulation (NMES), transcutaneous electrical nerve stimulation (TENS), and functional electrical stimulation (FES) [120]. NMES can activate type II fibers, which are most affected by aging and exhibit a decline in functional activity [121]. TENS has good therapeutic efficacy for refractory pain following PNI; moreover, non-invasive or minimally invasive TENS is readily accepted by patients. However, the effects of TENS are short-lived. Moreover, because elderly individuals experience reduced sensitivity to TENS, the therapeutic outcomes are often less effective compared to younger people [118].

It has been reported that peripheral nerves are highly sensitive to ultrasound stimulation, which can reversibly modulate nerve conduction [122]. Studies have also found that low-intensity pulsed ultrasound (LIPUS) can promote functional recovery in entrapment neuropathy [123,124]. In peripheral nerve regeneration, low-intensity pulsed ultrasound may increase neurotrophic factor levels, activate SCs, and stimulate the cell signaling pathways related to cellular activation and mitosis. Given its preclinical benefits and lack of observed side effects, low-intensity pulsed ultrasound shows promise as a potential clinical therapy following nerve surgery or injury.

## 8. Future Research Directions and Challenges

With advancing age, the regenerative capacity of peripheral nerves significantly declines [126]. This may be associated with multiple factors, including the reduced intrinsic regenerative ability of neurons, impaired supporting cell (such as SC) function, and reduced angiogenesis. How these changes interact to cause a decline in regenerative capacity is a key issue in current research. In addition, the extracellular matrix provides essential support and signaling during nerve regeneration. During aging, changes in the composition and structure of the extracellular matrix may impede axonal growth and guidance [78]. Understanding how these changes influence nerve regeneration is another major research focus. Aging is frequently accompanied by increased inflammatory responses, a state termed “inflammaging” [76]. Although appropriate inflammation is crucial for regeneration following nerve injury, excessive or persistent inflammation leads to tissue damage and impaired regeneration. Achieving a balance between the inflammatory response and nerve regeneration without causing further damage is a significant research challenge. Several molecules—including transforming growth factor-β (TGF-β), insulin-like growth factor 1 (IGF-1), and certain microRNAs (e.g., miR-21)—play important roles in aging and nerve regeneration [127]. Elucidating the specific mechanisms of these molecules in aging could facilitate the development of novel therapeutic strategies.

The aging process varies among individuals, influenced by multiple factors including genetics, environment, and lifestyle. Developing personalized treatment approaches will therefore be a significant challenge in the future. The application of precision medicine may help in designing effective strategies to promote nerve regeneration based on an individual’s specific aging characteristics. With advances in biomaterials and tissue engineering, developing scaffold materials that mimic the environment of a young nervous system to support and promote nerve regeneration in the elderly has emerged as a future research direction. These materials should be biocompatible and can actively interact with the aging environment within the body [128]. In addition, the application of gene editing technologies (e.g., CRISPR/Cas9) and stem cell therapies may offer new approaches to addressing the impact of aging on nerve regeneration. However, these technologies still face numerous challenges in clinical application, including safety, efficacy, and ethical concerns [129,130]. Research on nerve regeneration can involve multidisciplinary collaboration, including neuroscience, materials science, molecular biology, and clinical medicine. How to effectively integrate the latest advancements from these disciplines will also pose a significant challenge for future studies.

Although many preclinical studies have identified potential regenerative therapies, their application to elderly patients still faces numerous challenges. Firstly, age-related changes in pharmacokinetics and pharmacodynamics significantly alter drug distribution, metabolism, and clearance processes [104]. Elderly individuals commonly take multiple medications, which further increases the risk of adverse drug interactions and toxicity [131]. More importantly, few clinical trials specifically stratify results based on age, which limits the evidence base for treatment strategies for elderly patients [132]. These limitations all indicate that it is necessary to develop models suitable for different age groups and scientifically design clinical trials to ensure effective transformation.

Furthermore, most research on the mechanisms of aging-related peripheral nerve regeneration is performed using rodent models, which may not fully replicate the pathological and physiological conditions of humans. This is because, compared to rodents, human nerves are larger in diameter and their nerve regeneration takes longer, which may limit extrapolation of the axonal growth rate observed in experimental animals [133,134]. Additionally, the differences in immune system tissues and macrophage phenotypes among different species may further increase translation complexity, thus requiring the use of large animal models of appropriate age with clinically relevant research designs, in order to provide practical and effective assistance for clinical treatment methods [135,136].

In summary, although progress has been made in understanding how aging affects peripheral nerve regeneration, developing effective treatments still requires overcoming the key issues and challenges mentioned above. Advancing technologies may gradually resolve these problems in the future, offering new hope for treating aging-related nerve injuries.

## 9. Conclusions

Organism aging leads to multiple age-dependent diseases, while also affecting the nervous system [137,138,139]. Aging-induced reduction in peripheral nerve regeneration, alterations in physiological state, and persistent chronic inflammatory responses will further exacerbate these degenerative changes [140,141,142].

Aging does not operate through a single mechanism alone but rather exerts its effects by disrupting the coordinated regeneration network involving gene expression programs, SC plasticity, immune regulation, mitochondrial homeostasis, and the vascular–extracellular matrix microenvironment [143,144,145,146]. These interrelated changes include impaired activation of regeneration-related genes, accumulation of aging markers, persistent inflammatory responses, increased oxidative stress, and reduced intolerance of the regeneration microenvironment [147,148]. Importantly, in clinical situations, these molecular changes are further exacerbated by age-related complications (such as diabetes, vascular diseases, and sarcopenia), which collectively limit the window of opportunity for and effectiveness of nerve repair [149,150,151,152]. Current research on PNI treatment primarily focuses on stem cell therapies. These approaches target the nervous system microenvironment, reactivating the regenerative capacity of injured nerves through relevant molecular mechanisms. Some studies also introduce biomaterials, in which biocompatible materials (such as nerve guidance conduits) are used to replace autologous nerve grafts [153]. These conduits can be loaded with neurotrophic factors and drugs and implanted in the body, effectively treating peripheral nerve injuries [154]. Various antiaging drugs can be used in clinical practice to eliminate senescent cells and reduce inflammatory responses [155]. In addition, physical therapies (e.g., electrical stimulation and ultrasound) have shown considerable efficacy in treating the PNS in the elderly [134]. However, it is necessary to emphasize that most experimental conclusions are derived from preclinical studies on young animal models. There are still gaps in the research on physiological differences among different age groups, changes in drug pharmacokinetics, and multiple medication situations in elderly patients [156]. Therefore, the feasibility and long-term safety of the translation and application of these intervention measures still need to be further verified. Understanding the molecular and cellular interactions within the aging nerve microenvironment during regeneration is key to identifying essential and feasible therapeutic targets. Developing novel and promising treatments requires determining the key factors that enhance the regenerative capacity of the PNS in aged organisms. This will also provide valuable insights for assessing clinical responses. Furthermore, the integration of interdisciplinary technologies is also essential. Integrating neuroscience with diverse disciplines, including chemistry, materials science, pharmacology, and artificial intelligence, will help develop multi-level, multidimensional treatments for nerve injuries, leading to more precise and effective therapeutic strategies for patients. With the continuous advancement and clinical application of these technologies, a significant improvement in functional recovery after nerve injury may be achievable.

In conclusion, this review emphasizes that aging-associated peripheral nerve regeneration impairment is a systemic failure, rather than a single-pathway physiological defect. By integrating metabolic dysfunction, immune remodeling, cellular senescence, and microenvironmental changes into a unified conceptual framework, we highlight the necessity of shifting from reductionist explanations to treatment strategies based on network models. The theoretical advancements in aging biology and regenerative neuroscience now need to be translated into clinically meaningful interventions for elderly patients. However, there are still many uncertainties in this field. Current knowledge is mainly based on preclinical models, which may not fully simulate the complex physiology of the elderly, and standardized measurement methods for neurofunctional outcomes in elderly patients are still lacking. Future research should prioritize age-based experimental designs, longitudinal clinical studies, and combined intervention measures targeting multiple interrelated pathways. Solving these unresolved issues will not only help us gain a deeper understanding of the aging-associated decline in regeneration capacity but also facilitate the smooth development of treatment plans for peripheral nerve injuries in our aging societies. In addition, this review has some limitations that should be acknowledged. This is a narrative synthesis rather than a systematic review and thus did not follow the research selection process guided by PRISMA. Although a careful review of the relevant literature was conducted to provide a comprehensive conceptual overview, the included studies were not based on pre-defined systematic search criteria. Future systematic reviews and meta-analyses will help to quantitatively assess the strength of the evidence and further refine the proposed conceptual framework.

## Figures and Tables

**Table 1:** Strategies and mechanisms for regenerating the aging nervous system.

Strategy	Evidence Level	Mechanism	Limitations	Translational Readiness	Reference
Pharmacological	Young and aged animal models	mTOR inhibition or senescent cell clearance	Immunosuppression; delayed wound healing; off-target cytotoxicity; intermittent dosing uncertainty	Moderate	[94,95,96,97,98,99,100,101,102,103,104]
Stem cell–based	Young animal; limited aged models; early human feasibility	Axonal regeneration and neurotrophic support	Heterogeneity; poor engraftment; manufacturing variability	Moderate	[105,106,107,108,109,110,111,112]
Gene therapy	Preclinical animal models	Targeted genetic and epigenetic modulation	Delivery efficiency; immune response to viral vectors; cost	Low–moderate	[113,114,115,116]
Physical therapy	Young animal; small human studies	Neuromodulation via physical stimuli	Protocol standardization; durability of effects	High	[117,118,119,120,121,122,123,124]

## Data Availability

No new data were generated in this study. All data supporting the findings of this review are available in the cited references.

## References

[1] Burnett M.G., Zager E.L. (2004). Pathophysiology of peripheral nerve injury: A brief review. Neurosurg. Focus.

[2] Yang Y., Gu W., Xu S., Wang S., Shi H., Zhang L., Meng X.G., Hong F., Du Y. (2024). Treatment for peripheral nerve injury: A protocol for a systematic review and Bayesian network meta-analysis. BMJ Open.

[3] Lavorato A., Aruta G., De Marco R., Zeppa P., Titolo P., Colonna M.R., Galeano M., Costa A.L., Vincitorio F., Garbossa D. (2023). Traumatic peripheral nerve injuries: A classification proposal. J. Orthop. Traumatol..

[4] Kong J., Teng C., Liu F., Wang X., Zhou Y., Zong Y., Wan Z., Qin J., Yu B., Mi D. (2024). Enhancing regeneration and repair of long-distance peripheral nerve defect injuries with continuous microcurrent electrical nerve stimulation. Front. Neurosci..

[5] Wang E.W., Zhang J., Huang J.H. (2015). Repairing peripheral nerve injury using tissue engineering techniques. Neural Regen. Res..

[6] Sun J., Zeng Q., Wu Z., Li Z., Gao Q., Liao Z., Li H., Ling C., Chen C., Wang H. (2024). Enhancing intraneural revascularization following peripheral nerve injury through hypoxic Schwann-cell-derived exosomes: An insight into endothelial glycolysis. J. Nano Biotechnol..

[7] Fenrich K., Gordon T. (2004). Canadian Association of Neuroscience review: Axonal regeneration in the peripheral and central nervous systems--current issues and advances. Can. J. Neurol. Sci..

[8] Goldberg J.L., Barres B.A. (2000). The relationship between neuronal survival and regeneration. Annu. Rev. Neurosci..

[9] Mietto B.S., Mostacada K., Martinez A.M. (2015). Neurotrauma and inflammation: CNS and PNS responses. Mediat. Inflamm..

[10] Weng Y.L., Joseph J., An R., Song H., Ming G.L. (2016). Epigenetic regulation of axonal regenerative capacity. Epigenomics.

[11] Ma S., Fu X., Lyu J., Guo W., Wang J., Li J., Ying Z. (2025). IGFBP2-Ceramide Pathway Mediates Divergent Myelin Breakdown in the PNS and CNS Following Injury. J. Neurosci. Off. J. Soc. Neurosci..

[12] Kadyan P., Singh L. (2024). Harmaline attenuates chemotherapy-induced peripheral neuropathy: Modulation of Nrf-2 pathway and NK-1 receptor signaling. Neurosci. Lett..

[13] Lau K.H.V. (2019). Laboratory Evaluation of Peripheral Neuropathy. Semin. Neurol..

[14] Ding Z., Chen Y., Huang G., Liao R., Zhang H., Zhou S., Liu X. (2025). Global, regional, and national burden of neuroblastoma and peripheral nervous system tumours in individuals aged over 60 from 1990 to 2021: A trend analysis of global burden of disease study. J. Health Popul. Nutr..

[15] Jagga M., Lehri A., Verma S. (2011). Effect of aging and anthropometric measurements on nerve conduction properties-A review. J. Exerc. Sci. Physiother..

[16] Verdier V., Csardi G., de Preux-Charles A.S., Medard J.J., Smit A.B., Verheijen M.H., Bergmann S., Chrast R. (2012). Aging of myelinating glial cells predominantly affects lipid metabolism and immune response pathways. Glia.

[17] Verdu E., Navarro X. (1995). Degeneration and regeneration of the peripheral nervous system with aging. Rev. Neurol..

[18] Verdu E., Ceballos D., Vilches J.J., Navarro X. (2000). Influence of aging on peripheral nerve function and regeneration. J. Peripher. Nerv. Syst..

[19] Li N.Y., Ge J., Vorrius B., Akelman E., Chen Q. (2021). COBRE for Skeletal Health and Repair: The Impact of Aging on the Capacity for Peripheral Nerve Regeneration. R. I. Med. J..

[20] Kawakita S., Naito K., Kubota D., Ueno Y., Negishi-Koga T., Yamamoto Y., Suzuki T., Imazu N., Kawamura K., Hattori N. (2025). Glycoprotein 130 improves repressor element-1 silencing transcription factor-related axon regenerative capacity in peripheral nerves with aging. Mol. Med. Rep..

[21] Rawji K.S., Kaushik D.K. (2021). Editorial: Age-Related Neuroimmunology of Degeneration and Repair. Front. Aging Neurosci..

[22] Jonsson S., Wiberg R., McGrath A.M., Novikov L.N., Wiberg M., Novikova L.N., Kingham P.J. (2013). Effect of delayed peripheral nerve repair on nerve regeneration, Schwann cell function and target muscle recovery. PLoS ONE.

[23] Zhang H., Zhang Z., Lin H. (2023). Research progress on the reduced neural repair ability of aging Schwann cells. Front. Cell. Neurosci..

[24] Suthar H.R., Goplani V.L., Prajapati P., Panchal V. (2022). Influence of aging on nerve conduction properties in healthy individuals: A cross-sectional study. Natl. J. Physiol. Pharm. Pharmacol..

[25] Stakos D.A., Stamatelopoulos K., Bampatsias D., Sachse M., Zormpas E., Vlachogiannis N.I., Tual-Chalot S., Stellos K. (2020). The Alzheimer’s Disease Amyloid-Beta Hypothesis in Cardiovascular Aging and Disease: JACC Focus Seminar. J. Am. Coll. Cardiol..

[26] Yang S., Park J.H., Lu H.C. (2023). Axonal energy metabolism, and the effects in aging and neurodegenerative diseases. Mol. Neurodegener..

[27] Pinares-Garcia P., Stratikopoulos M., Zagato A., Loke H., Lee J. (2018). Sex: A Significant Risk Factor for Neurodevelopmental and Neurodegenerative Disorders. Brain Sci..

[28] Buck S.A., Steinkellner T., Aslanoglou D., Villeneuve M., Bhatte S.H., Childers V.C., Rubin S.A., De Miranda B.R., O’Leary E.I., Neureiter E.G. (2021). Vesicular glutamate transporter modulates sex differences in dopamine neuron vulnerability to age-related neurodegeneration. Aging Cell.

[29] Bianco A., Antonacci Y., Liguori M. (2023). Sex and Gender Differences in Neurodegenerative Diseases: Challenges for Therapeutic Opportunities. Int. J. Mol. Sci..

[30] Su Y., Huang M., Thomas A.G., Maragakis J., Huizar K.D.J., Zheng Y., Wu Y., Farah M.H., Slusher B.S. (2024). GCPII Inhibition Promotes Remyelination after Peripheral Nerve Injury in Aged Mice. Int. J. Mol. Sci..

[31] Canta A., Chiorazzi A., Carozzi V.A., Meregalli C., Oggioni N., Bossi M., Rodriguez-Menendez V., Avezza F., Crippa L., Lombardi R. (2016). Age-related changes in the function and structure of the peripheral sensory pathway in mice. Neurobiol. Aging.

[32] Helbing D.L., Kirkpatrick J.M., Reuter M., Bischoff J., Stockdale A., Carlstedt A., Cirri E., Bauer R., Morrison H. (2023). Proteomic analysis of peripheral nerve myelin during murine aging. Front. Cell Neurosci..

[33] Lee N., Kim S., Lee J. (2022). Impairment of peripheral nerve regeneration by insufficient activation of the HGF/c-Met/c-Jun pathway in aged mice. Heliyon.

[34] Deck M., Van Hameren G., Campbell G., Bernard-Marissal N., Devaux J., Berthelot J., Lattard A., Médard J.-J., Gautier B., Guelfi S. (2022). Physiology of PNS axons relies on glycolytic metabolism in myelinating Schwann cells. PLoS ONE.

[35] Sasaki Y., Hackett A.R., Kim S., Strickland A., Milbrandt J. (2018). Dysregulation of NAD(+) Metabolism Induces a Schwann Cell Dedifferentiation Program. J. Neurosci..

[36] Yuan X., Klein D., Kerscher S., West B.L., Weis J., Katona I., Martini R. (2018). Macrophage Depletion Ameliorates Peripheral Neuropathy in Aging Mice. J. Neurosci..

[37] Pan X., Zhu Q., Pan L.-L., Sun J. (2022). Macrophage immunometabolism in inflammatory bowel diseases: From pathogenesis to therapy. Pharmacol. Ther..

[38] Franceschi C., Garagnani P., Parini P., Giuliani C., Santoro A. (2018). Inflammaging: A new immune-metabolic viewpoint for age-related diseases. Nat. Rev. Endocrinol..

[39] Yousefzadeh M.J., Flores R.R., Zhu Y., Schmiechen Z.C., Brooks R.W., Trussoni C.E., Cui Y., Angelini L., Lee K.A., McGowan S.J. (2021). An aged immune system drives senescence and ageing of solid organs. Nature.

[40] Sorge R.E., Mapplebeck J.C., Rosen S., Beggs S., Taves S., Alexander J.K., Martin L.J., Austin J.S., Sotocinal S.G., Chen D. (2015). Different immune cells mediate mechanical pain hypersensitivity in male and female mice. Nat. Neurosci..

[41] Klein S.L., Flanagan K.L. (2016). Sex differences in immune responses. Nat. Rev. Immunol..

[42] Schumacher M., Hussain R., Gago N., Oudinet J.-P., Mattern C., Ghoumari A.M. (2012). Progesterone synthesis in the nervous system: Implications for myelination and myelin repair. Front. Neurosci..

[43] Marciano G., Siniscalchi A., Di Gennaro G., Rania V., Vocca C., Palleria C., Catarisano L., Muraca L., Citraro R., Evangelista M. (2024). Assessing Gender Differences in Neuropathic Pain Management: Findings from a Real-Life Clinical Cross-Sectional Observational Study. J. Clin. Med..

[44] Lorefice L., Pellecchia M.T. (2026). Frailty across Neurological Diseases: Why Sex and Gender Matter. Ann. Neurol..

[45] Opalach K., Rangaraju S., Madorsky I., Leeuwenburgh C., Notterpek L. (2010). Lifelong calorie restriction alleviates age-related oxidative damage in peripheral nerves. Rejuvenation Res..

[46] Jessen K.R., Mirsky R. (2016). The repair Schwann cell and its function in regenerating nerves. J. Physiol..

[47] Stoll G., Jander S., Myers R.R. (2002). Degeneration and regeneration of the peripheral nervous system: From Augustus Waller’s observations to neuroinflammation. J. Peripher. Nerv. Syst..

[48] Gu D., Xia Y., Ding Z., Qian J., Gu X., Bai H., Jiang M., Yao D. (2024). Inflammation in the Peripheral Nervous System after Injury. Biomedicines.

[49] Yao Y., Zhou Y., Zhang Z., Huang Y., Jiang T., Xia Y., Gu D., Gu X., Bai H., Jiang M. (2025). Investigating How Thbs4 Regulates Degeneration and Regeneration of the Peripheral Nerve. Biomedicines.

[50] Stoll G., Muller H.W. (1999). Nerve injury, axonal degeneration and neural regeneration: Basic insights. Brain Pathol..

[51] Martini R., Schachner M. (1988). Immunoelectron microscopic localization of neural cell adhesion molecules (L1, N-CAM, and myelin-associated glycoprotein) in regenerating adult mouse sciatic nerve. J. Cell Biol..

[52] Arthur-Farraj P.J., Latouche M., Wilton D.K., Quintes S., Chabrol E., Banerjee A., Woodhoo A., Jenkins B., Rahman M., Turmaine M. (2012). c-Jun reprograms Schwann cells of injured nerves to generate a repair cell essential for regeneration. Neuron.

[53] Chen Z.-L., Strickland S. (2003). Laminin gamma1 is critical for Schwann cell differentiation, axon myelination, and regeneration in the peripheral nerve. J. Cell Biol..

[54] Perry V.H., Brown M.C. (1992). Role of macrophages in peripheral nerve degeneration and repair. Bioessays.

[55] Gerdts J., Brace E.J., Sasaki Y., DiAntonio A., Milbrandt J. (2015). SARM1 activation triggers axon degeneration locally via NAD(+) destruction. Science.

[56] Coleman M.P., Hoke A. (2020). Programmed axon degeneration: From mouse to mechanism to medicine. Nat. Rev. Neurosci..

[57] Yuan Y., Wang Y., Wu S., Zhao M.Y. (2022). Review: Myelin clearance is critical for regeneration after peripheral nerve injury. Front. Neurol..

[58] Gomez-Sanchez J.A., Carty L., Iruarrizaga-Lejarreta M., Palomo-Irigoyen M., Varela-Rey M., Griffith M., Hantke J., Macias-Camara N., Azkargorta M., Aurrekoetxea I. (2015). Schwann cell autophagy, myelinophagy, initiates myelin clearance from injured nerves. J. Cell Biol..

[59] Perry V.H., Brown M.C., Gordon S. (1987). The macrophage response to central and peripheral nerve injury. A possible role for macrophages in regeneration. J. Exp. Med..

[60] Bruck W. (1997). The role of macrophages in Wallerian degeneration. Brain Pathol..

[61] Mosley K., Cuzner M.L. (1996). Receptor-mediated phagocytosis of myelin by macrophages and microglia: Effect of opsonization and receptor blocking agents. Neurochem. Res..

[62] Kuhlmann T., Wendling U., Nolte C., Zipp F., Maruschak B., Stadelmann C., Siebert H., Bruck W. (2002). Differential regulation of myelin phagocytosis by macrophages/microglia, involvement of target myelin, Fc receptors and activation by intravenous immunoglobulins. J. Neurosci. Res..

[63] Huang Y., Wu L., Zhao Y., Guo J., Li R., Ma S., Ying Z. (2024). Schwann cell promotes macrophage recruitment through IL-17B/IL-17RB pathway in injured peripheral nerves. Cell Rep..

[64] Varadarajan S.G., Hunyara J.L., Hamilton N.R., Kolodkin A.L., Huberman A.D. (2022). Central nervous system regeneration. Cell.

[65] Sprenger-Svacina A., Svacina M.K.R., Otlu H.G., Gao T., Sheikh K.A., Zhang G. (2025). Endoneurial immune interplay in peripheral nerve repair: Insights and implications for future therapeutic interventions. Front. Neurosci..

[66] Rios R., Jablonka-Shariff A., Broberg C., Snyder-Warwick A.K. (2021). Macrophage roles in peripheral nervous system injury and pathology: Allies in neuromuscular junction recovery. Mol. Cell Neurosci..

[67] Freria C.M., Bernardes D., Almeida G.L., Simoes G.F., Barbosa G.O., Oliveira A.L. (2016). Impairment of toll-like receptors 2 and 4 leads to compensatory mechanisms after sciatic nerve axotomy. J. Neuro Inflamm..

[68] Hsieh C.H., Rau C.S., Kuo P.J., Liu S.H., Wu C.J., Lu T.H., Wu Y.C., Lin C.W. (2017). Knockout of toll-like receptor impairs nerve regeneration after a crush injury. Oncotarget.

[69] Xia Y., Yao Y., Feng Y., Zhou Y., Jiang M., Ding Z., Qian J., Bai H., Cai M., Yao D. (2025). Toll-Like Receptor 4 (TLR4) Promotes DRG Regeneration and Repair after Sciatic Nerve Injury via the ERK-NF-kB Pathway. Mol. Neurobiol..

[70] Jessen K.R., Mirsky R. (2019). The Success and Failure of the Schwann Cell Response to Nerve Injury. Front. Cell Neurosci..

[71] Zhou X., Deng X., Liu M., He M., Long W., Xu Z., Zhang K., Liu T., So K.-F., Fu Q.-L. (2023). Intranasal delivery of BDNF-loaded small extracellular vesicles for cerebral ischemia therapy. J. Control Release.

[72] Zhou S., Shen D., Wang Y., Gong L., Tang X., Yu B., Gu X., Ding F. (2012). microRNA-222 targeting PTEN promotes neurite outgrowth from adult dorsal root ganglion neurons following sciatic nerve transection. PLoS ONE.

[73] Lopez-Leal R., Diaz-Viraque F., Catalan R.J., Saquel C., Enright A., Iraola G., Court F.A. (2020). Schwann cell reprogramming into repair cells increases miRNA-21 expression in exosomes promoting axonal growth. J. Cell Sci..

[74] Mehdipour M., Thakkar V., Chang S. (2025). Enhancing peripheral nerve regeneration in aging: The role of Schwann cells, c-Jun, and emerging therapeutic strategies. Geroscience.

[75] Terenghi G. (1999). Peripheral nerve regeneration and neurotrophic factors. J. Anat..

[76] Buttner R., Schulz A., Reuter M., Akula A.K., Mindos T., Carlstedt A., Riecken L.B., Baader S.L., Bauer R., Morrison H. (2018). Inflammaging impairs peripheral nerve maintenance and regeneration. Aging Cell.

[77] Wagstaff L.J., Gomez-Sanchez J.A., Fazal S.V., Otto G.W., Kilpatrick A.M., Michael K., Wong L.Y.N., Ma K.H., Turmaine M., Svaren J. (2021). Failures of nerve regeneration caused by aging or chronic denervation are rescued by restoring Schwann cell c-Jun. Elife.

[78] Maita K.C., Garcia J.P., Avila F.R., Torres-Guzman R.A., Ho O., Chini C.C.S., Chini E.N., Forte A.J. (2023). Evaluation of the Aging Effect on Peripheral Nerve Regeneration: A Systematic Review. J. Surg. Res..

[79] Yang Z.J., Zhao C.L., Liang W.Q., Chen Z.R., Du Z.D., Gong S.S. (2024). ROS-induced oxidative stress and mitochondrial dysfunction: A possible mechanism responsible for noise-induced ribbon synaptic damage. Am. J. Transl. Res..

[80] Masin L., Bergmans S., Van Dyck A., Farrow K., De Groef L., Moons L. (2024). Local glycolysis supports injury-induced axonal regeneration. J. Cell Biol..

[81] Biswas G., Anandatheerthavarada H.K., Zaidi M., Avadhani N.G. (2003). Mitochondria to nucleus stress signaling: A distinctive mechanism of NFkappaB/Rel activation through calcineurin-mediated inactivation of IkappaBbeta. J. Cell Biol..

[82] Coppé J.-P., Patil C.K., Rodier F., Sun Y., Muñoz D.P., Goldstein J., Nelson P.S., Desprez P.-Y., Campisi J. (2008). Senescence-associated secretory phenotypes reveal cell-nonautonomous functions of oncogenic RAS and the p53 tumor suppressor. PLoS Biol..

[83] Childs B.G., Gluscevic M., Baker D.J., Laberge R.M., Marquess D., Dananberg J., van Deursen J.M. (2017). Senescent cells: An emerging target for diseases of ageing. Nat. Rev. Drug Discov..

[84] Gavini C.K., Elshareif N., Aubert G., Germanwala A.V., Calcutt N.A., Mansuy-Aubert V. (2022). LXR agonist improves peripheral neuropathy and modifies PNS immune cells in aged mice. J. Neuro Inflamm..

[85] Kirkham P. (2007). Oxidative stress and macrophage function: A failure to resolve the inflammatory response. Biochem. Soc. Trans..

[86] Zhang Y., Zhao Q., Chen Q., Xu L., Yi S. (2023). Transcriptional Control of Peripheral Nerve Regeneration. Mol. Neurobiol..

[87] Aare S., Spendiff S., Vuda M., Elkrief D., Perez A., Wu Q., Mayaki D., Hussain S.N., Hettwer S., Hepple R.T. (2016). Failed reinnervation in aging skeletal muscle. Skelet. Muscle.

[88] Moradzadeh A., Borschel G.H., Luciano J.P., Whitlock E.L., Hayashi A., Hunter D.A., Mackinnon S.E. (2008). The impact of motor and sensory nerve architecture on nerve regeneration. Exp. Neurol..

[89] Callaghan B.C., Cheng H.T., Stables C.L., Smith A.L., Feldman E.L. (2012). Diabetic neuropathy: Clinical manifestations and current treatments. Lancet Neurol..

[90] Goncalves N.P., Vaegter C.B., Andersen H., Ostergaard L., Calcutt N.A., Jensen T.S. (2017). Schwann cell interactions with axons and microvessels in diabetic neuropathy. Nat. Rev. Neurol..

[91] Eftekharpour E., Fernyhough P. (2022). Oxidative Stress and Mitochondrial Dysfunction Associated with Peripheral Neuropathy in Type 1 Diabetes. Antioxid. Redox Signal.

[92] Sheikh A.M., Yano S., Tabassum S., Nagai A. (2024). The Role of the Vascular System in Degenerative Diseases: Mechanisms and Implications. Int. J. Mol. Sci..

[93] Larsson L., Degens H., Li M., Salviati L., Lee Y.I., Thompson W., Kirkland J.L., Sandri M. (2019). Sarcopenia: Aging-Related Loss of Muscle Mass and Function. Physiol. Rev..

[94] Johnson S.C., Rabinovitch P.S., Kaeberlein M. (2013). mTOR is a key modulator of ageing and age-related disease. Nature.

[95] Neff F., Flores-Dominguez D., Ryan D.P., Horsch M., Schröder S., Adler T., Afonso L.C., Aguilar-Pimentel J.A., Becker L., Garrett L. (2013). Rapamycin extends murine lifespan but has limited effects on aging. J. Clin. Investig..

[96] Carosi J.M., Sargeant T.J. (2019). Rapamycin and Alzheimer disease: A double-edged sword?. Autophagy.

[97] Li Z., Zhang Z., Ren Y., Wang Y., Fang J., Yue H., Ma S., Guan F. (2021). Aging and age-related diseases: From mechanisms to therapeutic strategies. Biogerontology.

[98] Arriola Apelo S.I., Lamming D.W. (2016). Rapamycin: An InhibiTOR of Aging Emerges From the Soil of Easter Island. J. Gerontol. A Biol. Sci. Med. Sci..

[99] Zhu Y., Tchkonia T., Pirtskhalava T., Gower A.C., Ding H., Giorgadze N., Palmer A.K., Ikeno Y., Hubbard G.B., Lenburg M. (2015). The Achilles’ heel of senescent cells: From transcriptome to senolytic drugs. Aging Cell.

[100] Talpaz M., Shah N.P., Kantarjian H., Donato N., Nicoll J., Paquette R., Cortes J., O’Brien S., Nicaise C., Bleickardt E. (2006). Dasatinib in imatinib-resistant Philadelphia chromosome-positive leukemias. N. Engl. J. Med..

[101] Porkka K., Koskenvesa P., Lundán T., Rimpiläinen J., Mustjoki S., Smykla R., Wild R., Luo R., Arnan M., Brethon B. (2008). Dasatinib crosses the blood-brain barrier and is an efficient therapy for central nervous system Philadelphia chromosome–positive leukemia. Blood J. Am. Soc. Hematol..

[102] Flanagan E.W., Most J., Mey J.T., Redman L.M. (2020). Calorie Restriction and Aging in Humans. Annu. Rev. Nutr..

[103] Redman L.M., Ravussin E. (2010). Caloric restriction in humans: Impact on physiological, psychological, and behavioral outcomes. Antioxid. Redox Signal..

[104] Mangoni A.A., Jackson S.H. (2004). Age-related changes in pharmacokinetics and pharmacodynamics: Basic principles and practical applications. Br. J. Clin. Pharmacol..

[105] Kizilay Z., Aktas S., Cetin N.K., Ilhan D.B., Ersoy G., Erken H.A. (2018). Effect of Systemic Application of Bone Marrow-Derived Mesenchymal Stem Cells on Healing of Peripheral Nerve Injury in an Experimental Sciatic Nerve Injury Model. Turk. Neurosurg..

[106] Wakitani S., Nawata M., Tensho K., Okabe T., Machida H., Ohgushi H. (2007). Repair of articular cartilage defects in the patello-femoral joint with autologous bone marrow mesenchymal cell transplantation: Three case reports involving nine defects in five knees. J. Tissue Eng. Regen. Med..

[107] Flores I., Blasco M.A. (2010). The role of telomeres and telomerase in stem cell aging. FEBS Lett..

[108] Baker N., Boyette L.B., Tuan R.S. (2015). Characterization of bone marrow-derived mesenchymal stem cells in aging. Bone.

[109] Wilson A., Shehadeh L.A., Yu H., Webster K.A. (2010). Age-related molecular genetic changes of murine bone marrow mesenchymal stem cells. BMC Genom..

[110] Zhang R.C., Du W.Q., Zhang J.Y., Yu S.X., Lu F.Z., Ding H.M., Cheng Y.B., Ren C., Geng D.Q. (2021). Mesenchymal stem cell treatment for peripheral nerve injury: A narrative review. Neural Regen. Res..

[111] Huang Z., Powell R., Phillips J.B., Haastert-Talini K. (2020). Perspective on Schwann Cells Derived from Induced Pluripotent Stem Cells in Peripheral Nerve Tissue Engineering. Cells.

[112] Nguyen H., Zarriello S., Coats A., Nelson C., Kingsbury C., Gorsky A., Rajani M., Neal E.G., Borlongan C.V. (2019). Stem cell therapy for neurological disorders: A focus on aging. Neurobiol. Dis..

[113] Wang W., Zheng Y., Sun S., Li W., Song M., Ji Q., Wu Z., Liu Z., Fan Y., Liu F. (2021). A genome-wide CRISPR-based screen identifies KAT7 as a driver of cellular senescence. Sci. Transl. Med..

[114] Chao C.-C., Shen P.-W., Tzeng T.-Y., Kung H.-J., Tsai T.-F., Wong Y.-H. (2021). Human iPSC-Derived Neurons as A Platform for Deciphering the Mechanisms behind Brain Aging. Biomedicines.

[115] Yi W., Wen Y., Tan F., Liu X., Lan H., Ye H., Liu B. (2019). Impact of NF-kappaB pathway on the apoptosis-inflammation-autophagy crosstalk in human degenerative nucleus pulposus cells. Aging.

[116] MacDonald J.L., Tharin S., Hall S.E. (2022). Epigenetic regulation of nervous system development and function. Neurochem. Int..

[117] Mat S., Tan M.P., Kamaruzzaman S.B., Ng C.T. (2015). Physical therapies for improving balance and reducing falls risk in osteoarthritis of the knee: A systematic review. Age Ageing.

[118] ElAbd R., Alabdulkarim A., AlSabah S., Hazan J., Alhalabi B., Thibaudeau S. (2022). Role of Electrical Stimulation in Peripheral Nerve Regeneration: A Systematic Review. Plast. Reconstr. Surg. Glob. Open.

[119] Al-Majed A.A., Neumann C.M., Brushart T.M., Gordon T. (2000). Brief electrical stimulation promotes the speed and accuracy of motor axonal regeneration. J. Neurosci..

[120] Ni L., Yao Z., Zhao Y., Zhang T., Wang J., Li S., Chen Z. (2023). Electrical stimulation therapy for peripheral nerve injury. Front. Neurol..

[121] Acaroz Candan S., Akoglu A.S., Bugusan S., Yuksel F. (2019). Effects of neuromuscular electrical stimulation of quadriceps on the quadriceps strength and functional performance in nursing home residents: A comparison of short and long stimulation periods. Geriatr. Gerontol. Int..

[122] Young R.R., Henneman E. (1961). Functional effects of focused ultrasound on mammalian nerves. Science.

[123] Mourad P.D., Lazar D.A., Curra F.P., Mohr B.C., Andrus K.C., Avellino A.M., McNutt L.D., Crum L.A., Kliot M. (2001). Ultrasound accelerates functional recovery after peripheral nerve damage. Neurosurgery.

[124] Crisci A.R., Ferreira A.L. (2002). Low-intensity pulsed ultrasound accelerates the regeneration of the sciatic nerve after neurotomy in rats. Ultrasound Med. Biol..

[125] Han B., Xi W., Hong Y., Gu L., Chao Y., Li L., Liu C., Yang L., Chao J., Yao H. (2022). Mutual regulation of noncoding RNAs and RNA modifications in psychopathology: Potential therapeutic targets for psychiatric disorders?. Pharmacol. Ther..

[126] Sutherland T.C., Geoffroy C.G. (2020). The Influence of Neuron-Extrinsic Factors and Aging on Injury Progression and Axonal Repair in the Central Nervous System. Front. Cell Dev. Biol..

[127] Wan T., Zhang F.S., Qin M.Y., Jiang H.R., Zhang M., Qu Y., Wang Y.L., Zhang P.X. (2024). Growth factors: Bioactive macromolecular drugs for peripheral nerve injury treatment-Molecular mechanisms and delivery platforms. Biomed. Pharmacother..

[128] Powell R., Eleftheriadou D., Kellaway S., Phillips J.B. (2021). Natural Biomaterials as Instructive Engineered Microenvironments That Direct Cellular Function in Peripheral Nerve Tissue Engineering. Front. Bioeng. Biotechnol..

[129] Caobi A., Dutta R.K., Garbinski L.D., Esteban-Lopez M., Ceyhan Y., Andre M., Manevski M., Ojha C.R., Lapierre J., Tiwari S. (2020). The Impact of CRISPR-Cas9 on Age-related Disorders: From Pathology to Therapy. Aging Dis..

[130] Yi S., Zhang Y., Gu X., Huang L., Zhang K., Qian T., Gu X. (2020). Application of stem cells in peripheral nerve regeneration. Burn. Trauma.

[131] Rodrigues M.C.S., Oliveira C.d. (2016). Drug-drug interactions and adverse drug reactions in polypharmacy among older adults: An integrative review. Rev. Lat. Am. Enferm..

[132] Zulman D.M., Sussman J.B., Chen X., Cigolle C.T., Blaum C.S., Hayward R.A. (2011). Examining the evidence: A systematic review of the inclusion and analysis of older adults in randomized controlled trials. J. Gen. Intern. Med..

[133] Fu S.Y., Gordon T. (1997). The cellular and molecular basis of peripheral nerve regeneration. Mol. Neurobiol..

[134] Gordon T. (2020). Peripheral Nerve Regeneration and Muscle Reinnervation. Int. J. Mol. Sci..

[135] Mestas J., Hughes C.C.W. (2004). Of mice and not men: Differences between mouse and human immunology. J. Immunol..

[136] Wynn T.A., Vannella K.M. (2016). Macrophages in Tissue Repair, Regeneration, and Fibrosis. Immunity.

[137] Fraser H.C., Kuan V., Johnen R., Zwierzyna M., Hingorani A.D., Beyer A., Partridge L. (2022). Biological mechanisms of aging predict age-related disease co-occurrence in patients. Aging Cell.

[138] Tenchov R., Sasso J.M., Wang X., Zhou Q.A. (2024). Aging Hallmarks and Progression and Age-Related Diseases: A Landscape View of Research Advancement. ACS Chem. Neurosci..

[139] Shimazu T., Tamura N., Shimazu K. (2005). Aging of the autonomic nervous system. Nihon Rinsho.

[140] Tanaka K., Webster H.D. (1991). Myelinated fiber regeneration after crush injury is retarded in sciatic nerves of aging mice. J. Comp. Neurol..

[141] Boss G.R., Seegmiller J.E. (1981). Age-related physiological changes and their clinical significance. West. J. Med..

[142] Garcia-Dominguez M. (2025). Pathological and Inflammatory Consequences of Aging. Biomolecules.

[143] Goldman J.A., Poss K.D. (2020). Gene regulatory programmes of tissue regeneration. Nat. Rev. Genet..

[144] Rankin M.M., Kushner J.A. (2010). Aging induces a distinct gene expression program in mouse islets. Islets.

[145] Reuter H., Perner B., Wahl F., Rohde L., Koch P., Groth M., Buder K., Englert C. (2022). Aging Activates the Immune System and Alters the Regenerative Capacity in the Zebrafish Heart. Cells.

[146] Wu N.N., Zhang Y., Ren J. (2019). Mitophagy, Mitochondrial Dynamics, and Homeostasis in Cardiovascular Aging. Oxid. Med. Cell Longev..

[147] Dimri G.P., Lee X., Basile G., Acosta M., Scott G., Roskelley C., Medrano E.E., Linskens M., Rubelj I., Pereira-Smith O. (1995). A biomarker that identifies senescent human cells in culture and in aging skin in vivo. Proc. Natl. Acad. Sci. USA.

[148] Dodig S., Cepelak I., Pavic I. (2019). Hallmarks of senescence and aging. Biochem. Med..

[149] Stout M.B., Justice J.N., Nicklas B.J., Kirkland J.L. (2017). Physiological Aging: Links Among Adipose Tissue Dysfunction, Diabetes, and Frailty. Physiology.

[150] Triposkiadis F., Xanthopoulos A., Parissis J., Butler J., Farmakis D. (2022). Pathogenesis of chronic heart failure: Cardiovascular aging, risk factors, comorbidities, and disease modifiers. Heart Fail. Rev..

[151] Yoo S.Z., No M.H., Heo J.W., Park D.H., Kang J.H., Kim S.H., Kwak H.B. (2018). Role of exercise in age-related sarcopenia. J. Exerc. Rehabil..

[152] Ebenezer G.J., O’Donnell R., Hauer P., Cimino N.P., McArthur J.C., Polydefkis M. (2011). Impaired neurovascular repair in subjects with diabetes following experimental intracutaneous axotomy. Brain.

[153] Gaudin R., Knipfer C., Henningsen A., Smeets R., Heiland M., Hadlock T. (2016). Approaches to Peripheral Nerve Repair: Generations of Biomaterial Conduits Yielding to Replacing Autologous Nerve Grafts in Craniomaxillofacial Surgery. Biomed. Res. Int..

[154] Vijayavenkataraman S. (2020). Nerve guide conduits for peripheral nerve injury repair: A review on design, materials and fabrication methods. Acta Biomater..

[155] Pessoa J., Nobrega-Pereira S., de Jesus B.B. (2024). Senescent cell-derived vaccines: A new concept towards an immune response against cancer and aging?. Aging.

[156] Mitchell S.J., MacArthur M.R., Kane A.E. (2025). Optimizing preclinical models of ageing for translation to clinical trials. Br. J. Clin. Pharmacol..

